# The MOZ-BRPF1 acetyltransferase complex in epigenetic crosstalk linked to gene regulation, development, and human diseases

**DOI:** 10.3389/fcell.2022.1115903

**Published:** 2023-01-11

**Authors:** Tiina Viita, Jacques Côté

**Affiliations:** St-Patrick Research Group in Basic Oncology, Oncology Division of Centre Hospitalier Universitaire de Québec-Université Laval Research Center, Laval University Cancer Research Center, Quebec City, QC, Canada

**Keywords:** MOZ, KAT6A, BRPF1, epigenetics, development, cancer, MYST acetyltransferase, MORF

## Abstract

Acetylation of lysine residues on histone tails is an important post-translational modification (PTM) that regulates chromatin dynamics to allow gene transcription as well as DNA replication and repair. Histone acetyltransferases (HATs) are often found in large multi-subunit complexes and can also modify specific lysine residues in non-histone substrates. Interestingly, the presence of various histone PTM recognizing domains (reader domains) in these complexes ensures their specific localization, enabling the epigenetic crosstalk and context-specific activity. In this review, we will cover the biochemical and functional properties of the MOZ-BRPF1 acetyltransferase complex, underlining its role in normal biological processes as well as in disease progression. We will discuss how epigenetic reader domains within the MOZ-BRPF1 complex affect its chromatin localization and the histone acetyltransferase specificity of the complex. We will also summarize how MOZ-BRPF1 is linked to development *via* controlling cell stemness and how mutations or changes in expression levels of MOZ/BRPF1 can lead to developmental disorders or cancer. As a last touch, we will review the latest drug candidates for these two proteins and discuss the therapeutic possibilities.

## Introduction

The human genome comprises of approximately three billion nucleotides of DNA and for this reason the DNA must be highly compacted inside the nucleus (International Human Genome Sequencing Consortium, 2004). This is primarily done by wrapping DNA around histone proteins, to form the basic unit of chromatin, i.e., the nucleosome. DNA holds the genetic information needed for gene expression, which is essential for cell functions. As the DNA is tightly wrapped around histones in the form of chromatin, dynamic changes in the chromatin structure are necessary for effective gene expression, DNA repair as well as DNA replication. Epigenetic factors such as ATP-dependent chromatin remodeling complexes and histone modifying complexes modulate the compaction of genomic loci, allowing activation and repression of genes at given regions on the chromosome. The histone modifying complexes are responsible of histone post-transitional modifications (PTMs), which are deposited on the histone tails by specific enzymes (known as writers) and mark specific loci in the genome. Local specific combinations of these PTMs form epigenetic signatures that can be recognized by effector proteins (readers). As most PTMs are reversible, various enzymes (erasers) can also remove these marks. Chromatin modifying complexes are large multifunctional protein complexes and the presence of reader modules in these complexes ensures their specific localization, enabling the epigenetic crosstalk and context-specific activity [reviewed in (Talbert and Henikoff, 2021; Millan-Zambrano et al., 2022)].

One group of chromatin modifying complexes are histone acetyltransferases (HATs), mainly responsible of acetylating histone tails while some of them also have non-histone targets (Steunou et al., 2014). Acetylation of histone lysine residue removes the positive charge of this residue, which weakens the interactions between histones and DNA as well as between nucleosomes, leading to a more open chromatin structure. Open chromatin is more accessible to the transcription machinery; therefore, acetylation correlates with transcriptionally active genes. Acetylated lysine residues can also be recognized by proteins containing various reader domains, which makes them binding sites for other regulatory factors, including other chromatin modifying or remodeling complexes. Beside transcription, histone acetylation is also linked with DNA repair (Murr et al., 2006; Lashgari et al., 2022) and it has been shown to regulate the activity of DNA replication origins (Lubelsky et al., 2014). Different HATs can be categorized to different families based on their homologies: the Gcn5-related acetyltransferase (GNAT) family [reviewed in (Vetting et al., 2005)], the p300/CBP family [reviewed in (Dancy and Cole, 2015)], and the MYST family (named after its founding members, MOZ, Ybf2/Sas3, Sas2, and Tip60) [reviewed in (Avvakumov and Cote, 2007)]. In this review, we will focus on the 4-subunit MOZ-BRPF1 acetyltransferase complex, which belongs to MYST acetyltransferase family. We will summarize how different binding motifs inside the subunits of the complex drive the chromatin localization and biological functions of this histone modifying complex. We will also discuss how this complex has been linked to development as well as disease and hypothesize the therapeutic possibilities of drug candidates that target its structure/function.

### MYST-family acetyltransferase MOZ forms a 4-subunit complex *via* interaction with BRPF1

Monocytic leukemia zinc finger protein (MOZ, also known as KAT6A) is a MYST family acetyltransferase, which was first discovered as a fusion partner of CBP in acute myeloid leukemia (AML) patients (Borrow et al., 1996). It is closely related to MORF (monocytic leukemia zinc finger protein related factor, also known as KAT6B), which shares 60% homology (Champagne et al., 1999). MOZ/MORF enzymes are found part of 4-subunit complexes that include ING4/5 (inhibitor of growth 4 or 5), MEAF6 (mammalian Esa1-associated factor-6) and BRPF1 (bromodomain and PHD finger-containing protein 1), the latter acting as scaffolding subunit binding simultaneously to MOZ or MORF on one hand, and ING4/5 and MEAF6 on the other (Doyon et al., 2006; Ullah et al., 2008; Klein et al., 2014; Feng et al., 2016; Klein et al., 2019). Although MOZ and MORF have high similarity and the same binding partners, they seem to drive different biological processes. MORF is required for neurogenesis and cerebral formation (Thomas et al., 2000; Merson et al., 2006; Sheikh et al., 2012), whereas knockout studies in mice have shown that MOZ is essential for normal hematopoiesis (Katsumoto et al., 2006; Thomas et al., 2006). These functional differences between MOZ and MORF may be due to distinct spatiotemporal gene expression, non-histone targets or regulation by post-translational modifications.

### The MOZ/MORF histone acetyltransferase has multiple functional domains

**FIGURE 1:**
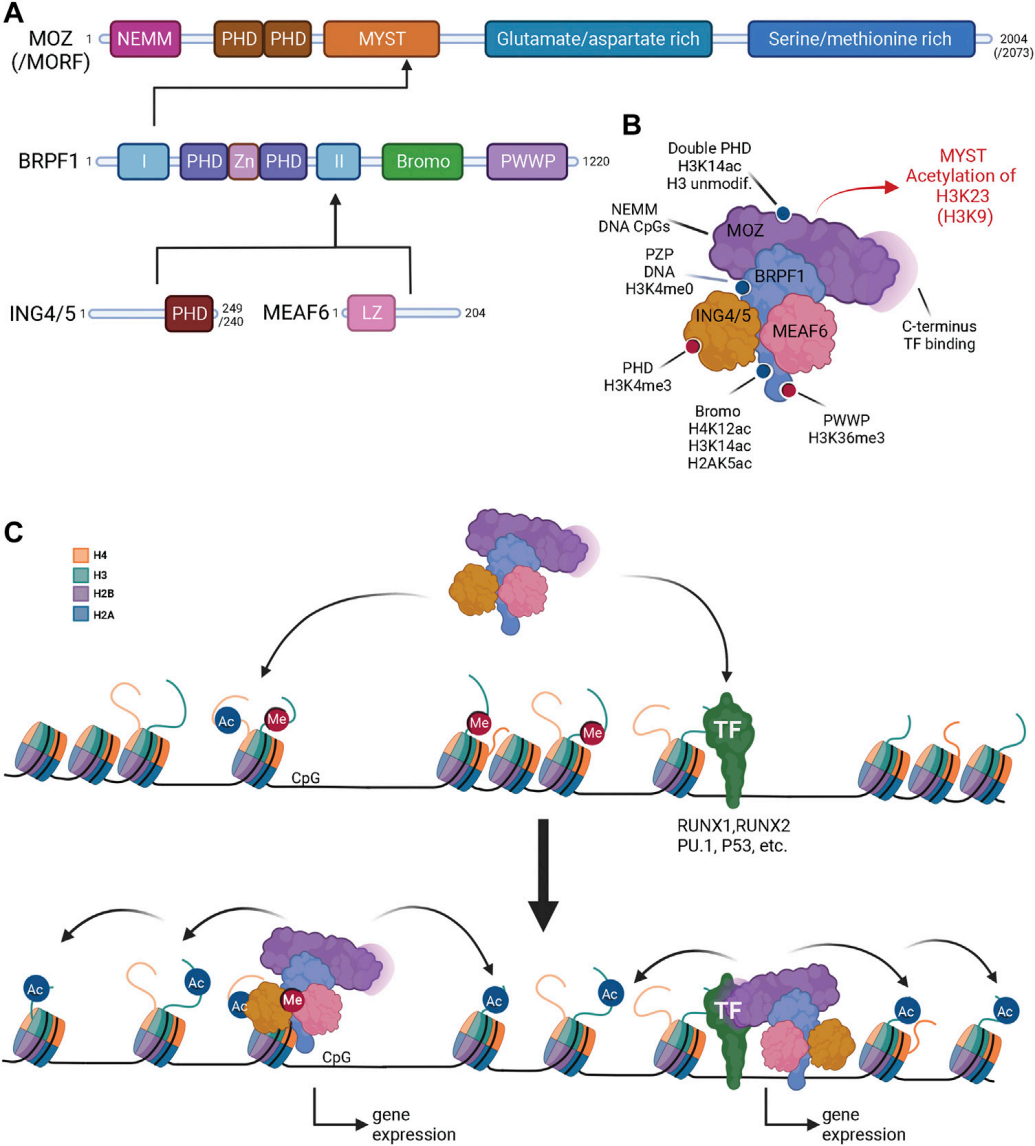
Multiple chromatin binding domains in the 4-subunit MOZ-BRPF1 complex inform about its biological properties and importance in transcription activation. **(A)** Protein domains of the MOZ-BRPF1 complex; MOZ, BRPF1, ING4/5 and MEAF6. Domains labelled as follows: NEMM, N-terminal part of Enok, MOZ and MORF; PHD, plant homeodomain-linked zinc finger; MYST, MYST histone acetyltransferase domain; Zn, zinc knuckle; I and II, Epc-homology region I and II; Bromo, bromo domain; PWWP, proline-tryptophan-tryptophan-proline-containing domain; LZ leucine zipper. Numbers correspond to total residues that each protein possesses. MOZ paralog MORF is indicated in parenthesis as it has the same domain structures/arrangement. **(B)** Predicted MOZ-BRPF1 complex orientation. Scaffolding subunit BRPF1 connects MOZ to ING4/5 and MEAF6. Experimentally determined histone marks/chromatin and DNA binding sites of the MOZ-BRPF1 complex are indicated. Red dots represent methylated histones and blue dots acetylated histones. Read arrow indicates acetyltransferase substrates of the complex. TF, transcription factor. **(C)** The MOZ-BRPF1 complex in gene activation. The complex binds to active transcription sites *via* acetylated H4 and/or methylated H3K4 and H3K36 and/or unmethylated CpG islands (left) or *via* transcription factors (TF) (right). After binding to specific chromatin regions, MOZ acetylates targeted histone lysines, which leads to opening of chromatin and induced gene expression. Individual histones in nucleosomes are indicated with different colors (upper left corner). DNA is represented by the black line Created with BioRender.com.

MOZ and MORF proteins harbor various domains [NEMM, double plant homeodomain (PHD) finger [DPF], MYST, glutamate/aspartate-rich and serine/methionine-rich domain] (Figure 1A) (Champagne et al., 1999; Champagne et al., 2001). The C-terminal region of MOZ/MORF contains the glutamate/aspartate-rich and serine/methionine-rich domains. These domains have been linked with transcription activation (Champagne et al., 1999; Champagne et al., 2001). This is supported by studies showing the serine/methionine-rich domain can bind and activate transcription factors RUNX1, RUNX2 and TP53 (Kitabayashi et al., 2001a; Pelletier et al., 2002; Rokudai et al., 2009). Moreover, these two domains are often lost in leukemic protein fusions with MOZ/MORF produced by chromosomal translocations (Borrow et al., 1996; Carapeti et al., 1998; Chaffanet et al., 2000), which indicates that lack of these domains may deregulate the MOZ-dependent gene expression network, leading to cancer. The NEMM (N-terminal part of Enok, MOZ or MORF) domain is also found in Enok, the homologous protein in *Drosophila melanogaster* (Huang et al., 2016). This domain had not been well characterized while showing similarities with the winged helix of linker histones H1 and H5 and being implicated in the nuclear localization of MOZ (Kitabayashi et al., 2001a). Overexpression studies with truncated constructs have suggested that the N-terminal region (1–84) of MOZ can interact with RNA polymerase 2 (RNAPII) (Miyamoto et al., 2020). Most recently, this domain was shown to recognize unmethylated CpG islands on DNA, *in vitro* and *in vivo*, and to be required for recruitment of MOZ/MORF and its HAT activity on the genome (Becht et al., 2023). Winged helix 1 (WH1) is critical for this function, recognizing unmethylated CpGs, and cooperates with winged helix 2 (WH2) to bind nucleosomes.

The most studied domains of MOZ/MORF are the DPF and MYST domains in the N-terminal part of the proteins. The DPF domain was first found to interact with the N-terminal part of histone H3 tail and helps the protein to localize to chromatin. Surprisingly, DPF interaction with the H3 tail strengthens when either H3K9 or H3K14 are acetylated but is crippled if H3K4 is modified/methylated (Ali et al., 2012) (Figure 1B). It seems that H3R2 must also be unmodified for DPF to bind H3K14ac (Qiu et al., 2012). Further structural studies have shown that DPF with the help of the MYST domain induces an α-helical conformation of H3K4-T11, which revealed a unique way of H3 recognition (Dreveny et al., 2014). It was suggested that this induced helical structure explains how MOZ recognizes the H3K4 methylation status and facilitates its own binding to selected chromatin regions. More recently, biochemical, structural and ChIP data argued that DPF allows a specific cross-talk *in vivo*, recognizing H3K14ac deposited by another HAT, HBO1/KAT7, to induce local H3K23 acetylation by MOZ/MORF (Klein et al., 2019). Importantly, DPF can associate with different forms of lysine acylation on histones and has a strong preference for H3K14 crotonylation (H3K14cr) (Tan et al., 2011; Xiong et al., 2016; Klein et al., 2017).

The first *in vitro* HAT assays with the MYST domain of MOZ showed acetylation of free histones H3, H4 and H2A, but it was not able by itself to acetylate nucleosomes (Kitabayashi et al., 2001a; Champagne et al., 2001) (Figure 1B). Structural studies with MOZ MYST domain revealed that it contains more divergent N- and C-terminal regions and possesses helix-turn-helix (HTH) DNA-binding motifs that can enhance catalytic activity (Holbert et al., 2007). Interestingly, a small region at the C-terminus of the MYST domain is needed to associate with BRPF1, the scaffolding platform of the 4-subunit complex (Ullah et al., 2008). Binding to BRPF1 enables MOZ to acetylate histone H3 tails within nucleosomes. BRPF1 also enhanced the transcription coactivator activity of MOZ, which indicates that complex formation helps MOZ to bind to specific genomic regions to activate transcription. As mentioned above, MOZ/MORF acetylates H3K23 and can be influenced by DPF recognition of H3K14 acylation (Klein et al., 2019). Interestingly, a recent study suggests that MOZ could prefer to propionylate this histone residue (Yan et al., 2017; Yan et al., 2020).

It has been suggested that a small region after the MYST domain of MOZ could bind to ENL, a common MLL protein fusion partner in mixed linage leukemia (Miyamoto et al., 2020). As this interaction was observed in chromatin fractions, it remains to be verified if ENL binds directly to MOZ or if they only occupy the same chromatin regions, as native purification from soluble nuclear extracts did not detect such interaction (Feng et al., 2016; Klein et al., 2019). Additionally, it has been suggested that MOZ would directly interact with MLL itself, so ENL could also associate with MOZ *via* MLL interaction (Paggetti et al., 2010; Miyamoto et al., 2020), or that ENL acts downstream of MOZ through recognition by its YEATS domain of H3K9ac deposited by the HAT (Yan et al., 2022). It is important to note that NuA3, the homologous HAT complex in budding yeast, was shown to associate with TAF14, a YEATS domain-containing protein that also recognizes H3K9ac (John et al., 2000; Shanle et al., 2015).

### BRPF1 is the scaffold responsible for the MOZ complex assembly and enables binding to nucleosomes

As mentioned above, BRPF1 is the scaffolding subunit of MOZ/MORF acetyltransferase complexes binding simultaneously MOZ or MORF, ING4/5 and MEAF6 (Figure 1A) (Ullah et al., 2008; Klein et al., 2014; Feng et al., 2016; Klein et al., 2019). This protein also has multiple functional domains such as a PHD-Zn knuckle-PHD (PZP) domain, a bromodomain and a chromo/Tudor-related Pro-Tyr-Tyr-Pro (PWWP) domain.

BRPF1 has been shown to able to bind both MOZ/MORF and HBO1 through its N-terminal region (domain I) (Ullah et al., 2008; Lalonde et al., 2013) but purification in native conditions indicates that the vast majority of BRPF1 is associated with MOZ/MORF *in vivo* (Feng et al., 2016). A small region between the PZP and bromodomain is required for association of ING4/5 and MEAF6 (Ullah et al., 2008; Lalonde et al., 2014) (domain II) (Figure 1A). Other domains of BRPF1 are epigenetic reader domains, which allow the protein to interact with chromatin. While BRPF1 PWWP domain is not important for acetyltransferase activity (Lalonde et al., 2013), it can recognize the H3K36me3 histone mark that is present on transcribed regions (Vezzoli et al., 2010) and seems required for BRPF1 to associate with chromatin regions *in vivo* (Laue et al., 2008). BRPF1 bromodomain can recognize histone acetylation marks including H2AK5ac, H4K12ac, H4K8ac, H4K5ac, and H3K14ac (Poplawski et al., 2014) (Figure 1B). It seems to have highest affinity towards H2AK5ac, followed by H4K12ac and H3K14ac. The PZP domain in BRPF1 is highly conserved in BRPF2/3 and JADE1/2/3 proteins associated with HBO1 complexes (Saksouk et al., 2009; Mishima et al., 2011; Avvakumov et al., 2012; Feng et al., 2016). Biochemical and structural studies with BRPF1 PZP domain indicate that both PHDs are required for the MOZ-BRPF1-ING5-MEAF6 complex to bind histone H3 *in vivo* and acetylate chromatin substrates *in vitro*. PHD2 assists PHD1 by allowing tight binding to a nucleosome with extra-nucleosomal linker DNA, while PHD1 locks in histone H3 N-terminus (Lalonde et al., 2013; Klein et al., 2016; Klein et al., 2020). Interestingly, the extreme N-terminus of BRPF1 also seems to have a role in the acetylation of chromatin substrates, driving the histone H3 tail specificity (Lalonde et al., 2013).

### ING4/5 and MEAF6 subunits link MOZ to the H3K4me3 mark near transcription start sites

Although ING4/5 and MEAF6 do not directly bind to MOZ (Figure 1A), they were found to increase and specify the acetyltransferase activity of MOZ when compared to the MOZ-BRPF1 dimer (Ullah et al., 2008). ING4/5 proteins also contain a PHD domain specific for the H3K4me3 mark found around the transcription start sites of genes *in vitro* and *in vivo* (Shi et al., 2006; Champagne et al., 2008; Lalonde et al., 2013). Furthermore, the HAT activity of the ING5-containing MOZ/MORF complexes is greatly stimulated on H3K4me3 peptides when compared with unmodified or H3K9me peptides (Lalonde et al., 2013). This suggests that the PHD domain of ING4/5 not only guides the MOZ/MORF-BRPF1 complexes to active transcription sites, but it also enhances the acetyltransferase activity of the complexes at these sites.

MEAF6 function is much less understood while conserved from yeast to humans, essential for cell viability (depmap.org) and stoichiometric subunit of MOZ, MORF, HBO1 and NuA4/TIP60 HAT complexes. However, its role in the MOZ-BRPF1 complex does not seem to be as important as ING4/5 (Doyon et al., 2006; Ullah et al., 2008). Structural studies showed that MEAF6 associates with ING proteins and domain II of the scaffold subunit through three intertwined helices (Xu et al., 2016). Further studies are needed to understand the function of this small subunit in HAT complexes containing ING subunits.

### The MOZ-BRPF1 complex in biological processes and disease

The biochemical properties of the MOZ-BRPF1 acetyltransferase complex have shown that it has multiple chromatin binding/reader domains and several mechanisms for its recruitment to chromatin have been proposed. For example, ING4/5 and BRPF1 seem to recruit the complex to active transcription sites *via* binding to H3K4me3 (Lalonde et al., 2013) or H3K36me3 (Vezzoli et al., 2010), whereas the C-terminal part of MOZ has been shown to associate with different transcription factors (Kitabayashi et al., 2001a; Pelletier et al., 2002; Rokudai et al., 2009; Aikawa et al., 2010) possibly targeting the complex to a small subset of specific genes (Figure 1C). Being able to recognize specific histone PTMs indicates that localization of the MOZ-BRPF1 complex is regulated by other histone modifying complexes, e.g. HBO1-dependent H3K14ac recognized by MOZ DPF reader domain (Klein et al., 2019). This diversity of possible recruitment/regulatory mechanisms has made it difficult to fully understand the biological functions of this complex. Therefore, it has been important to conduct knockdown and knockout experiments of individual complex partners *in vivo* to pinpoint their actual role in biological processes. Careful examination of the biological processes will also help to identify how these complexes might behave in different pathological situations like cancer.

### The MOZ-BRPF1 complex has an essential role in development *via* regulation of *HOX* genes and cell stemness

**FIGURE 2:**
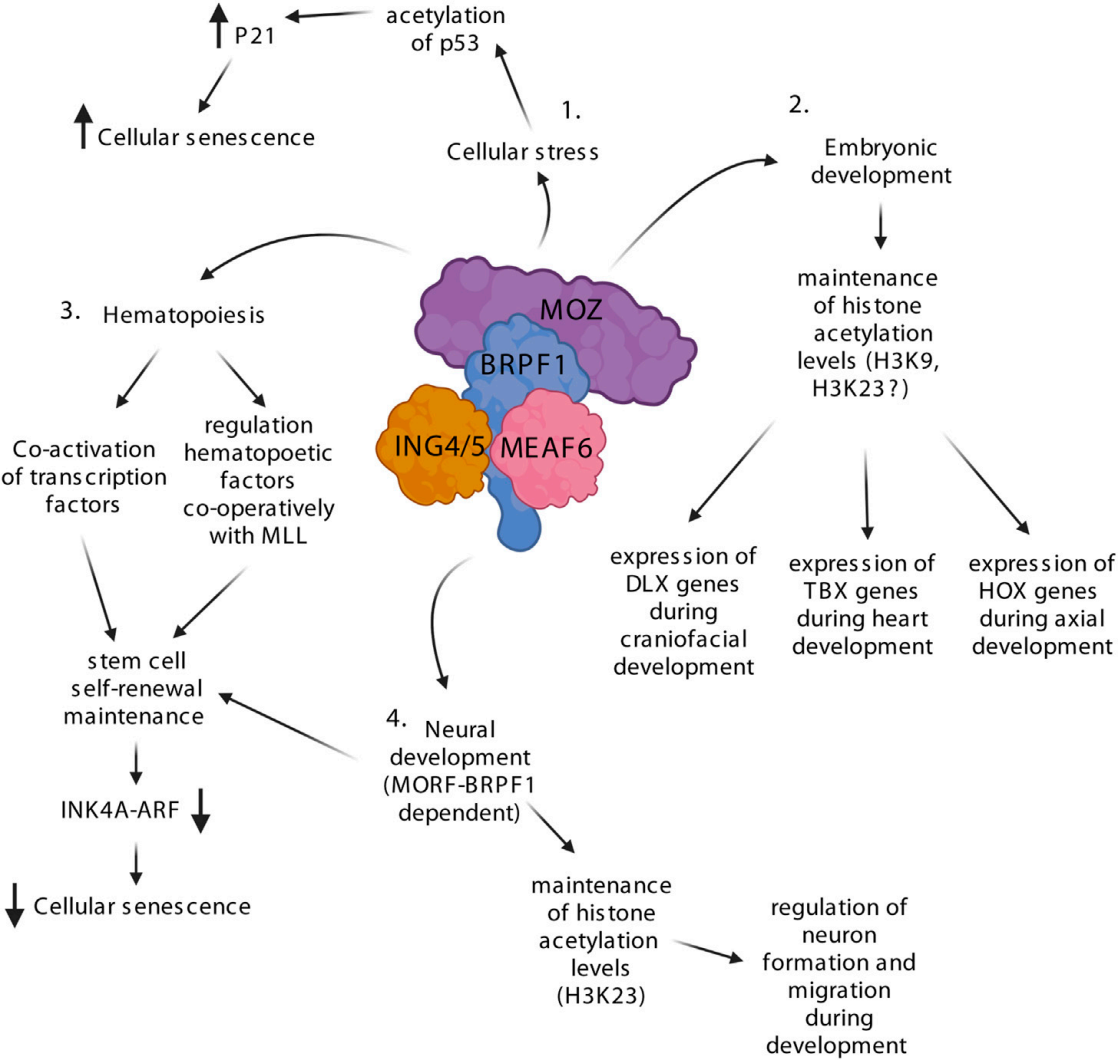
The MOZ-BRPF1 complex regulates various steps of development *via* controlling H3 acetylation levels. 1) Upon cellular stress, MOZ drives cellular senescence by interacting with PML-p53, which enhances p53 acetylation leading to expression of the p21 senescence factor. 2) MOZ is involved in embryogenesis by controlling the expression of multiple essential transcription factor families such as homeobox (HOX), T-box (TBX) and distal-less homeobox (DLX). Knockout experiments with MOZ show a decrease of H3K9ac levels at these genes, suggesting that the MOZ-BRPF1 complex regulates H3K9ac during development. H3K23ac is labeled with a question mark since its levels have not been fully addressed during MOZ KO studies. 3) MOZ and BRPF1 are crucial regulators of hematopoietic and neuronal stem cells and precursors. The MOZ-BRPF1 complex inhibits premature senescence of stem cells by downregulating the INK4a/ARF pathway. 4. Depletion of BRPF1 leads to decreased formation and motion of neurons causing neurodevelopmental defects. Loss of H3K23ac in the dorsal cortex of BRPF1 KO mice indicates that MOZ-BRPF1 or MORF-BRPF1 regulate this acetylation mark during neuronal development. Created with BioRender.com.

The first MOZ knockout studies were done in zebrafish, where MOZ was found to be an important regulator of developmental *HOX* genes (Miller et al., 2004; Crump et al., 2006) (Figure 2). *HOX* gene clusters are known to encode a family of transcriptional regulators that elicit distinct developmental programs along the head-to-tail axis of animals (Mallo and Alonso, 2013). Depletion of MOZ leads to defects in the zebrafish facial skeleton and lowers the expression levels of *HOXB* genes, which are known to be crucial regulators of head formation in zebrafish (Miller et al., 2004; Crump et al., 2006). Acetylation of histones by MOZ seems to be important in *HOX* gene regulation as inhibition of histone deacetylases (HDACs) rescues the phenotype. Mechanistic studies pinpointed that MOZ supports the skeletal development in the head (Crump et al., 2006). BRPF1 knockout studies in zebrafish follow the same trend as MOZ, which suggests that BRPF1 does not have MOZ-independent functions in skeletal development (Laue et al., 2008; Hibiya et al., 2009). This mechanism seems to be conserved as defects in craniofacial and caudal skeletons were also found in MOZ KO mice (Hibiya et al., 2009; Voss et al., 2009). In mice, MOZ seems to have a global effect on *Hox* gene expression and specification of the anterior expression boundary of *Hox* genes (*Hoxc8/9*). MOZ depletion diminishes the H3K9 acetylation mark of specific *Hox* genes in chromatin immunoprecipitation (ChIP) assays, without affecting the global levels of H3K9Ac (Voss et al., 2009). Thus, the MOZ-BRPF1 complex seems to regulate the transcription of *HOX* genes *via* H3K9 acetylation. Interestingly, ING5 association to HOX genes seems to be dependent on MOZ, suggesting that the complex is recruited *via* MOZ. It has also been shown that MOZ and BMI1, which is part of polycomb repressive complex 1 (PRC1), work simultaneously during the regulation of *Hox* genes in axial skeleton development (Sheikh et al., 2015a). Deletion of *Moz* or *Bmi1* lead to opposing defects in axial skeleton development. However, if both are mutated, the mice shows a morphologically normal axial skeleton. Further studies showed that these two complexes indeed have opposing roles as PRC1 is required to prevent premature activation of *Hox* genes, while MOZ-BRPF1 is needed to activate and maintain normal levels of *Hox* gene expression after the initial activation phase. The role of MOZ in craniofacial development has been studied with full and conditional *Moz* knockout (KO) mice (Voss et al., 2012; Vanyai et al., 2019). Studies with the conditional KO showed that MOZ occupies and regulates H3K9 acetylation levels at distal-less homeobox (DLX) transcription factor-encoding *Dlx5* gene loci and lowered expression of all DLX family genes and their targets (Vanyai et al., 2019). While conditional KO embryos showed downregulation of DLX transcription factors, there was upregulation of Runx2 and other bone-specific downstream effectors possibly leading to premature osteoblast lineage differentiation, causing craniofacial anomalies. Both DLX5 and MOZ have been previously linked with the RUNX2 transcription factor (Pelletier et al., 2002; Lee et al., 2003), leading Vanyai et al. (2019) to suggest a molecular mechanism where MOZ together with DLX5 regulate osteoblast development *via* suppression of RUNX2. Studies with the KO mice also showed that MOZ is involved in the development of the palate, facial structures, and is also linked to defects in the thymus and the cardiovascular system (Voss et al., 2012). Interestingly, the defects in the cardiovascular system closely resemble those seen in the DiGeorge syndrome (DGS). DGS is caused by mutations in the T-box 1 (*TBX1*) gene and further studies concluded that MOZ regulates *TBX1*, *TXB2* and *TXB5* expression *via* maintaining the H3K9ac levels at their loci. MOZ was shown to activate *TBX1* and *TBX5* in mesodermal cells during heart development (Vanyai et al., 2015).

As MOZ was first found as a fusion partner of CBP produced by chromosomal translocation found in AML patients (Borrow et al., 1996), it suggested that it may be involved in hematopoiesis (Figure 2). Indeed, two independent knockout studies in mice showed that MOZ depletion impairs hematopoiesis and causes embryonic lethality around E14.5 (Katsumoto et al., 2006) or just before birth (Thomas et al., 2006), depending on the knockout mice strain. Different phenotypic analyses revealed that, although the precursors for all hematopoietic lineages and hematopoietic stem cells (HSCs) were present in MOZ KO mice, their amount and functionalities were dramatically reduced (Katsumoto et al., 2006; Thomas et al., 2006). Similar defects were found in mice carrying a point mutation in the catalytic site of MOZ, indicating that the acetyltransferase activity is required for efficient hematopoiesis (Perez-Campo et al., 2009). In parallel, BRPF1 has an important role in MOZ-mediated acetylation in HSCs as its depletion leads to dramatic reduction of H3 acetylation in bone marrow (You et al., 2016). MOZ has also been shown to be needed for B cell proliferation (Sheikh et al., 2015b). Moreover, conditional MOZ KO mice have reduced immune response and alterations in memory development of B cells (Good-Jacobson et al., 2014) and T cells (Newman et al., 2016). The role of MOZ in hematopoiesis is also supported by earlier biochemical studies showing that it can interact and activate hematopoietic transcription factors RUNX1 and PU.1 (Kitabayashi et al., 2001a; Bristow and Shore, 2003; Katsumoto et al., 2006). Mechanistically, MOZ seems to regulate important hematopoietic factors such as *HOX* genes, MIP-1α, c-Mpl, Meis1 and c-Kit as they are downregulated in KO/KD mice (Katsumoto et al., 2006; Sheikh et al., 2015b).

MOZ has been implicated with histone methyltransferase MLL in the regulation of *HOX* genes in hematopoietic cells (Paggetti et al., 2010). MLL is known to be important for HSC self-renewal (McMahon et al., 2007) and it regulates *HOX* genes by methylating H3K4, which promotes active transcription (Milne et al., 2002). In human cord blood cells, MOZ and MLL bind to the same promoter regions of various *HOX* genes and deletion of one or the other leads to lower levels of acetylated H3 as well as methylated H3K4 (Paggetti et al., 2010). These findings suggest a positive feedback loop where MOZ-mediated H3 acetylation favors MLL-dependent H3K4 methylation and *vice versa*, leading to increased transcription. This is supported in part by the fact that pre-acetylated H3 is a better substrate for methylation by MLL *in vitro* (Milne et al., 2002; Jain et al., 2022). Since most of the downregulated factors, including MLL, are linked to self-renewal and repopulating potential of HSCs, MOZ has likely a more profound role in the expansion and proliferation of early hematopoietic precursors. Indeed, MOZ can prevent HSCs and mouse embryonic fibroblasts (MEFs) from entering senescence *via* regulation of the INK4a/ARF pathway (Perez-Campo et al., 2014; Sheikh et al., 2015c). Upon MOZ depletion, the INK4a/ARF pathway is upregulated and studies in MEFs showed that MOZ maintains H3K9ac levels and expression of the INK4a/ARF pathway inhibitors CDC6, EZH2 and E2F2 (Sheikh et al., 2015c). In a related context, MOZ has already been linked to stress-induced cellular senescence, as it regulates expression of p21 and increases premature senescence through acetylation of TP53 (Rokudai et al., 2009; Rokudai et al., 2013). Thus, MOZ seems to either activate or prevent cellular senescence depending on the context. In addition, MOZ has also been implicated in maintaining the quiescence of adult HSCs (Sheikh et al., 2016; Sheikh et al., 2017), a mechanism essential to prevent HSCs exhaustion that could lead to long-term loss of stem cells. MLL and MOZ likely work again together in regulating the HSC quiescence as MLL is required to self-renew and maintain a non-cycling population of adult HSCs (Jude et al., 2007).

The knockout studies in zebrafish demonstrated that depletion of either MOZ or BRPF1 leads to similar defects and decreased in *HOX* gene expression, suggesting strictly overlapping roles in that organism (Miller et al., 2004; Crump et al., 2006; Laue et al., 2008; Hibiya et al., 2009). However, BRPF1 KO mice suffer more severe developmental issues and earlier embryonic lethality around E9.5 (You et al., 2014). During embryonic development, high BRPF1 expression is seen in the neuroepithelial cells of the neural tube and the headfold (You et al., 2014) while MOZ is expressed at low levels in these regions (Thomas et al., 2006). These findings indicate that BRPF1 could have MOZ independent functions during development. Indeed, BRPF1 KO mice embryos show various defects that are not seen in the MOZ KO (You et al., 2015a). Early embryonic lethality of the BRPF1-depleted mice can be explained by the blood vessel defects in placenta, which interrupt the nutrient and gas exchange between the embryonic and maternal circulation system. Also, the neurodevelopmental defects in the BRPF1 KO embryos are severe as the neural tube remains open (Figure 2). Immunostaining showed that formation and migration of neurons are compromised in the mutant embryos. Moreover, these defects are due to cell proliferation issues rather than apoptosis or DNA damage. Abnormal neuronal migration and cell cycle progression is also observed during brain and hippocampus development when BRPF1 is specifically depleted from the forebrain (bKO) (You et al., 2015b; You et al., 2015c). Behavioral tests with the BRPF1 bKO indicate that the mice suffer neocortical development problems, which commonly result in poor muscle motor coordination and learning disabilities. These findings indicate that BRPF1 has a crucial role in neuronal development. MOZ and MORF have been shown to regulate neuronal stem cell renewal (Sheikh et al., 2012; Perez-Campo et al., 2014) and loss of MORF causes neurological defects in mice (Thomas et al., 2000). Therefore, Since BRPF1 forms independent HAT complexes with MOZ and MORF, and possibly a small population of HBO1, its loss impacts the function of both (Doyon et al., 2006; Ullah et al., 2008; Lalonde et al., 2013; Feng et al., 2016; Klein et al., 2019). Based on biochemical studies BRPF1-MOZ/MORF prefers to acetylate H3K23, whereas BRPF1-HBO1 complex could acetylate H3K14 (Kueh et al., 2011; Lalonde et al., 2013; Feng et al., 2016; Yan et al., 2017; Klein et al., 2019). Accordingly, bone marrow cells of BRPF1 KO mice show lower levels of H3 acetylation at lysines 9, 14, and 23 (You et al., 2016). Although a small population of HBO1 could associate with BRPF1, it strongly prefers BRPF2/3 as binding partners, and loss of BRPF2 or BRPF3 have been linked with total loss of H3K14ac (Mishima et al., 2011; Feng et al., 2016; Yan et al., 2016). Moreover, studies with BRPF1 KO and heterozygote knockdown mice have shown total loss or reduced levels of H3K23ac in multiple tissues, indicating that BRPF1 solely governs specific H3K23 acetylation *via* MOZ/MORF in different tissues and cells (Yan et al., 2017). Therefore, loss of BRPF1 affects embryogenesis *via* preventing MOZ and MORF to acetylate H3K23.

In summary, while both MOZ and BRPF1 are important factors during embryogenesis (Figure 2), loss of BRPF1 creates more severe defects because of its function to enable chromatin binding and acetylation by both HAT enzymes. While MOZ binding to specific transcription factors allows the recruitment of the MOZ-BRPF1 complex to specific genomic loci, BRPF1 and ING4/5 are needed for efficient DNA and histone binding on local chromatin and acetyltransferase activity. A reduction in H3K9ac at specific target loci is observed in MOZ KO mice (Voss et al., 2009; Sheikh et al., 2015c; Vanyai et al., 2019). This is in contrast to biochemical and KD experiments in cells indicating that H3K23 is the major acetylation target of MOZ (Feng et al., 2016; Lv et al., 2017; Yan et al., 2017; Klein et al., 2019). Most recently, reduced H3K23 propionylation was also reported in MOZ KO embryo, indicating that MOZ can also performed other forms of lysine acylation *in vivo* (Yan et al., 2020), but H3K9 was not measured. Interestingly, antibody staining showed high H3K23ac in the E13.5 embryo liver and heart, whereas H3K23 propionylation (H3K23pr) was high in liver and cerebrocortical neuroepithelium. Although these two histone marks are chemically similar and linked with active transcription (Sabari et al., 2017), further studies are needed to address the role of them during development. Importantly, H3K23ac is by far one of the most abundant acetylation site in mammals [along H3K14ac and H4K16ac (Zheng et al., 2013)] and tumor suppressor TRIM24 has been shown to specifically bind H3K23ac with its bromodomain (Tsai et al., 2010). Therefore, it will be interesting to investigate its function alongside MOZ-BRPF1 complex in development. Additionally, studies are also needed to precisely investigate how and when MOZ and MORF may cooperate or play redundant roles during development.

### Mutations in MOZ and BRPF1 are linked to developmental disorders

**FIGURE 3:**
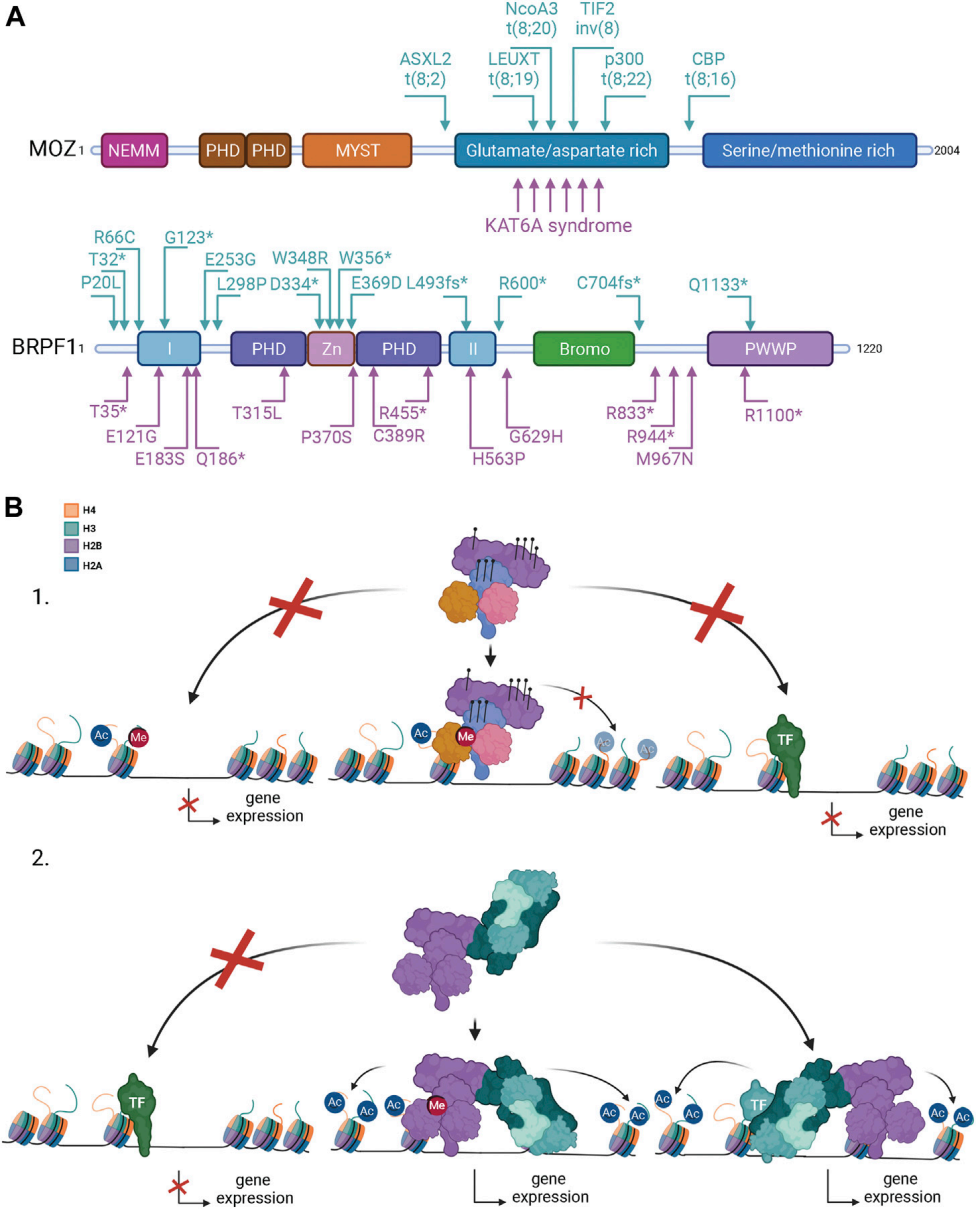
Mutations in MOZ and BRPF1 lead to developmental disorders and different forms of cancer. **(A)** MOZ is recurrently mutated in leukemia, non-hematologic malignancies, and developmental disorders with the common characteristics of intellectual disability and developmental delays. Arrows above the MOZ protein structure point to leukemia-associated translocations, the corresponding fusion partners being indicated. KAT6A syndrome refers to MOZ-specific intellectual disability disorders. For simplicity, only a few arrows below are used to illustrate mutation positions related to the KAT6A syndrome [refer to published reports for complete list of mutations (Arboleda et al., 2015; Tham et al., 2015; Kennedy et al., 2019)]. BRPF1 mutations are linked to developmental delays and cancer. Arrows above the BRPF1 protein structure indicate mutations associated with cancer (Huether et al., 2014; Kool et al., 2014; Aiello et al., 2019). Arrows below BRPF1 indicate mutations from patients with developmental disorders [modified from (Yan et al., 2020)]. Used abbreviations: CBP, CREB-binding protein; p300, E1A-associated p300 kDa protein; TIF2, transcription intermediary factor 2, ASXL2, additional sex combs-like 2; NcoA3, nuclear receptor co-activator three; leucine twenty homeobox, LEUTX. fs, reading frame shift; *, translational termination. Domain abbreviations for MOZ and BRPF1 are as in Figure 1. **(B)** Possible mechanisms how MOZ-BRPF1 mutations and translocations can change gene expression. 1) Mutations in the epigenetic reader domains of MOZ or BRPF1 can prevent chromatin binding on specific regions and alter gene expression (left). Mutations in the MOZ MYST domain or BRPF1 PZD domain can decrease or block the acetyltransferase activity of the complex leading to repressing chromatin structure unavailable for transcription (middle). Mutations in the C-terminal part of MOZ disrupts binding of specific (light green) transcription factors (TFs) blocking activation of genes (right). 2) Rearragements of the *MOZ* gene fuse the N-terminal of MOZ (indicated in A) with a fusion partner. Loss of MOZ C-terminus disrupts the complex binding to specific TFs (light green) blocking gene activation (left). The MOZ fusion protein can form a functional complex and binds to active transcription sites *via* BRPF1 and ING4/5 (middle). This can cause overexpression or silencing of target genes depending on the fusion partner. The MOZ complex can be recruited to fusion partner specific chromatin sites *via* TFs (teal) or other mechanisms leading to abnormal histone acetylation and gene expression in these sites (right) Created with BioRender.com.

Interestingly, patients with neurodevelopmental disorders have been shown to have mutations in MOZ (Arboleda et al., 2015; Tham et al., 2015; Kennedy et al., 2019) and BRPF1 (Mattioli et al., 2017; Yan et al., 2017; Naseer et al., 2020) (Figure 3A). Neurodevelopmental disorders impair or alter the growth and development of the brain and the central nervous system (Parenti et al., 2020). Transcriptional regulators or chromatin remodelers are often seen to be mutated in patients with neurodevelopmental disorders. Mutations in MOZ are usually heterozygous *de novo* mutations and they cause a disorder called the KAT6A syndrome (also known as Arboleda-Tham syndrome) (Arboleda et al., 2015; Tham et al., 2015). The core features of the syndrome are impaired intellectual development, significant speech/language deficit, heart defects, hypotonia, distinct facial features and gastrointestinal complications. Mechanistically, it was shown that H3K9ac is decreased in the fibroblasts collected from the patients, which is consistent with the KO/KD mice studies (Voss et al., 2009; Sheikh et al., 2015b). Truncating, frameshift, and non-sense mutations are the most common mutations in KAT6A syndrome patients, but missense mutations and mutations occurring at splice sites have also been mapped in some patients (Tham et al., 2015; Millan et al., 2016; Kennedy et al., 2019). Mutations in the MYST domain are rare as they are mostly found in those exons that encode the glutamate/aspartate-rich and serine/methionine-rich domains (Tham et al., 2015; Kennedy et al., 2019). Remarkably, patients with distal exon (16–18) mutations exhibit more severe symptoms than those with mutations in more proximal exons 1–15 (Kennedy et al., 2019). These findings indicate that the C-terminal part of MOZ, which is responsible for binding various transcription factors (Kitabayashi et al., 2001a; Pelletier et al., 2002; Katsumoto et al., 2006; Rokudai et al., 2009; Rokudai et al., 2013), is crucial for proper development in humans. Interestingly, while these monoallelic mutations in MOZ cause developmental anomalies (Tham et al., 2015; Kennedy et al., 2019), similar phenotypes are not observed in mice heterozygous MOZ KO (Voss et al., 2012; Vanyai et al., 2015; Vanyai et al., 2019). This suggests that the truncated MOZ mutant acts as dominant-negative. Another thing that needs to be pointed out is that the patients do not suffer from hematological abnormalities, although MOZ has an especially important role regulating the hematopoietic stem cells in mice (Katsumoto et al., 2006; Thomas et al., 2006). These issues require further studies to be fully understood. Interestingly, mutations in MORF, the MOZ paralog, have been linked to distinct developmental disorders with broad clinical spectrum called KAT6B disorders, which include the Genitopatellar syndrome and the Say-Barber-Biesecker-Young-Simpson syndrome (Zhang et al., 2020).

BRPF1 mutations are linked to patients showing intellectual developmental disorder with dysmorphic facies and ptosis (IDDDFP) (Mattioli et al., 2017; Yan et al., 2017; Demeulenaere et al., 2019; Pode-Shakked et al., 2019; Naseer et al., 2020). IDDDFP is an autosomal dominant neurodevelopmental disorder characterized by delayed motor and language development, intellectual disability, and dysmorphic facial features. The defects are milder but consistent with the BRPF1 KO studies in mice, underlining the importance of BRPF1 during neurodevelopment (You et al., 2015a; You et al., 2015b; You et al., 2015c). Similar to MOZ mutations, patients with BRPF1 mutations do not show hematological abnormalities (Mattioli et al., 2017; Yan et al., 2017; Demeulenaere et al., 2019; Pode-Shakked et al., 2019; Naseer et al., 2020; Yan et al., 2020), although loss of BRPF1 leads to acute bone-marrow failure in mice (You et al., 2016). In addition, while patients carry monoallelic mutations, no obvious phenotypes have been observed in heterozygous BRPF1 KO mice (You et al., 2015a; You et al., 2015b; You et al., 2015c). C-terminal truncating mutations are the most common ones and patients with truncations that disrupt MOZ/MORF binding or the PZP domain are considered the most severe (Mattioli et al., 2017; Yan et al., 2017; Demeulenaere et al., 2019; Pode-Shakked et al., 2019; Naseer et al., 2020; Yan et al., 2020). Biochemical and *in vivo* studies showed that disorder-linked BRPF1 mutations in the PZP domain block MOZ-dependent H3K23 acetylation (Mattioli et al., 2017; Yan et al., 2017). Slight decrease of H3K23ac was also seen in the histones extracted from patient fibroblasts, while global H3 acetylation levels is unchanged (Mattioli et al., 2017). Interestingly, lower H3K23ac levels are also observed in heterozygous BRPF1 KO mice, supporting the concept that heterozygous BRPF1 PZP mutations in humans can decrease H3K23ac levels mediated by MOZ and MORF (Yan et al., 2017).

### The MOZ-BRPF1 complex in cancer

All subunits of the MOZ-BRPF1 complex have been linked to tumorigenesis. ING4/5 are part of ING family of tumor suppressors also implicated in TP53 function (Dantas et al., 2019). Translocations within the *MEAF6* gene are linked with endometrial stromal sarcoma (Antonescu et al., 2014; Micci et al., 2014). BRPF1 has been implicated as tumor suppressor in childhood leukemia (Huether et al., 2014), prostate cancer (Sole et al., 2020) and medulloblastoma (Kool et al., 2014). Recently, truncated BRPF1, which is found in human medulloblastoma patients, was shown to promote the medulloblastoma formation with SmoM2 from postmitotic neurons in adult mice (Aiello et al., 2019). Since the BRPF1 expression levels are significantly lower in medulloblastoma when compared to normal tissue, it supports a tumor suppressor function. In contrast, BRPF1 is frequently overexpressed in hepatocellular carcinoma (HCC) (Cheng et al., 2021). Inhibition and knockout studies with BRPF1 in HCC showed that its overexpression induces tumor migration and proliferation, acting like an oncogene in this specific cancer. Thus, the potential role of BRPF1 needs to be addressed individually between different cancers.

Although all subunits of the MOZ-BRPF1 complex have been linked to cancer, prevalent studies are related to the functions of MOZ in leukemia. In hematologic malignancies, MOZ has been frequently shown to form fusion proteins produced by chromosomal translocations (Figure 3A). Identified fusion partners for MOZ are CREB binding protein (CBP) (Borrow et al., 1996), E1A Binding Protein P300 (p300) (Chaffanet et al., 2000; Kitabayashi et al., 2001b), transcriptional intermediary binding factor 2 (TIF2) (Carapeti et al., 1998), additional sex combs-like 2 (ASXL2) (Imamura et al., 2003), nuclear receptor co-activator 3 (NcoA3) (Esteyries et al., 2008) and leucine Twenty Homeobox (LEUTX) (Chinen et al., 2014; Sramkova et al., 2020; Panagopoulos et al., 2021). MOZ fusions are mostly found in AML, which originates from primitive populations of HSCs and progenitor cells (Roussel et al., 2020). In normal hematopoiesis, these primitive populations represent a suppressed cell reservoir, which are needed for production of mature circulating blood cells. However, genetic alterations can either reignite the self-renewal capacity of the progenitors or induce uncontrolled self-renewal of the HSCs. Predominantly, these genetic alterations cause abnormal accumulation of non-differentiating cells (leukemic stem cells, LSCs) in the bone marrow, which is diagnosed as AML. Among these fusion proteins, MOZ-TIF2 has been studied the most, shown to have transforming activity in cultured cells and to induce AML in mice (Deguchi et al., 2003). MOZ-TIF2 transforming activity relies on the fusion protein ability to recruit CBP *via* TIF2 to MOZ specific binding sites. MOZ DNA binding capacity through its HTH motifs seemed to be crucial for the ability of MOZ-TIF2 to induce AML in mice (Deguchi et al., 2003). However, the double mutation Q654E/G657E in MOZ acetyl-CoA binding site prevents the formation of AML (Shima et al., 2014). This indicates that DNA binding as well as histone acetyltransferase activity are required for inducing AML in mice. In addition, the translocation hotspot of MOZ is in the glutamate/aspartate-rich domain after the MYST HAT domain (Figure 3A). Therefore, the MOZ fusion proteins retain the N-terminal moiety of the protein, containing NEMM, DPF and MYST domains. Biochemical studies have shown that the MOZ-TIF2 fusion can form the functional complex with BRPF1, MEAF6 and ING4/5 (Ullah et al., 2008). Association with BRPF1 enables transcription activation and induced colony formation by MOZ-TIF2 (Shima et al., 2014). This indicates that complex formation is required for leukemic activity of the fusion proteins (Figure 3B). MOZ-TIF2 uses the same INK4a/ARF pathway to escape senescence as MOZ (Largeot et al., 2016) and it can also deplete CBP from PML bodies to inhibit transcriptional activity of p53 and the retinoic acid receptor RAR (Kindle et al., 2005; Collins et al., 2006). Later studies have shown that MOZ-TIF2 can promote self-renewal of the committed progenitor cells, which allows preleukemic expansion of cells in the bone marrow facilitating the development of AML (Huntly et al., 2004). MOZ-TIF2 transforming potential can be partly suppressed by knocking down the hematopoietic transcription factor PU.1 (Aikawa et al., 2010). These results support the idea that MOZ fusion proteins can promote self-renewal to produce LSCs. Gene expression profiling of MOZ-CBP AML patients showed that this fusion produces a pattern of *HOX* expression characteristic of self-renewal, with upregulation of *HOXA9*, *HOXA10*, cofactor *MEIS1* and marked downregulation of other homeobox genes (Camos et al., 2006). This profile partially resembles that of AML with MLL rearrangement, which may indicate that MLL and MOZ fusion proteins share gene targets for uncontrolled self-renewal. A recent study with MOZ and MLL fusions support this idea, showing that MOZ fusions and MLL can be recruited to unmethylated CpG-rich promoters to induce expression of leukemic genes (Miyamoto et al., 2020). In MLL-rearranged leukemia, MLL fusions rely on the AF4-ENL-P-TEFb complex (AEP/super elongation complexes) for transcriptional activation and DOT1L for transcriptional maintenance to fully transform hematopoietic progenitors to LSCs (Okuda et al., 2017). Miyamoto et al. (2020) claim that, in MOZ-rearranged leukemia, MLL is the recruiter of MOZ to specific genes as inhibition of MLL or DOT1L complex decreases the self-renewal capacity of leukemic stem cells produced by MOZ-TIF2. Mechanistically, the MOZ-CBP fusion seems to function in a slightly different manner, while both induce self-renewal capacity of cells (Kitabayashi et al., 2001a; Camos et al., 2006; Chan et al., 2007). While MOZ fusions drive leukemia by promoting self-renewal capacity of LSCs in different ways, implicating MLL, recent work demonstrates that MLL fusions themselves require endogenous MOZ function to induce AML, in part through ENL H3K9ac reader (Katsumoto et al., 2022; Yan et al., 2022). Cooperation between MOZ and MLL in oncogenic pathways has also been uncovered in gastrointestinal stromal tumors (Hemming et al., 2022).

In addition to leukemia, MOZ have been found abnormally expressed in other cancer types such as glioblastoma (Lv et al., 2017), medulloblastoma (Northcott et al., 2009), breast cancer (Yu et al., 2017), colorectal cancer (Mohammadi et al., 2019; Xie et al., 2020), uterine cervix cancer (Zack et al., 2013; Jiang et al., 2020), ovarian cancer (Zack et al., 2013; Liu et al., 2021), lung cancer, bladder cancer and several adenocarcinomas (Jiang et al., 2020). In most cancers MOZ expression is amplified. For example, lymphoma progression is highly dependent on MOZ expression, as depletion of one MOZ allele in mice can arrest transplantation-driven lymphoma development (Sheikh et al., 2015b). Recent studies on glioblastoma multiforme (GBM), ovarian, colorectal and breast cancers have dissected the molecular mechanisms used by MOZ to promote proliferation and growth of the tumors (Turner-Ivey et al., 2014; Lv et al., 2017; Yu et al., 2017; Lian et al., 2020; Xie et al., 2020; Liu et al., 2021). MOZ gene is frequently overexpressed in breast cancer samples, correlated with lower survival rates (Turner-Ivey et al., 2014; Yu et al., 2017; Jiang et al., 2020). MOZ is overexpressed in estrogen receptor-positive breast cancers, where it binds and regulates estrogen receptor α expression (Yu et al., 2017). SUM-52 breast cancer cells are known to have MOZ gene amplification (8p11-p12 amplicon), leading to its overexpression (Turner-Ivey et al., 2014). Downregulation of MOZ in these breast cancer cells inhibits tumor growth, supporting its function as an oncogene. Analysis of GBM primary specimens also linked MOZ overexpression with tumor progression and poor prognosis (Lv et al., 2017). MOZ depletion in GBM cell lines showed that high expression increases cell proliferation, migration, colony formation and tumor growth. Mechanistically, MOZ upregulates *PIK3CA* gene expression in GBM cells, activating the PI3K/ATK signaling pathway. MOZ binds to the *PIK3CA* promoter and acetylates H3K23, resulting in the recruitment of H3K23ac-specific reader/transcription factor TRIM24 (Lv et al., 2017). Interestingly, another study on colorectal cancer suggested a similar molecular mechanism but linked to YAP transcription/signaling (Xie et al., 2020). In colorectal cancer, MOZ cooperates with the DANCR long non-coding RNA (lncRNA) to bind and acetylate H3K23 at the *YAP* promoter, leading to the recruitment of TRIM24. Upregulation of *YAP* enhances proliferation and colony formation. YAP is part of the Hippo signaling pathway and regulates several biological processes including cell proliferation, organ size and cell fate (Pocaterra et al., 2020). Most recently, high expression of MOZ correlates with poor prognosis in ovarian cancer (Liu et al., 2021). MOZ was shown to promote tumor formation and drug resistance by acetylating the E3 ubiquitin ligase COP1. Normally, COP1 ubiquitinates β-catenin and regulates its degradation. Acetylation of COP1 impairs β-catenin ubiquitination, leading to its stabilization and abnormal activation of its signaling pathway, increasing proliferation and metastasis of ovarian cancer cells. Altogether, these studies have implicated MOZ in several crucial oncogenic pathways. It also becomes apparent that TRIM24 binding to the H3K23ac histone mark deposited by the MOZ-BRPF1 complex may become an important step in these oncogenic processes.

### MOZ-BRPF1 as potential therapeutic targets

Nowadays epigenetics plays a central role in many types of diseases, including cardiovascular diseases, neurological diseases, metabolic disorders and cancer. Because of the reversible nature of epigenetic events, scientists have seen epigenetic factors as potential drug targets for therapies. MOZ and BRPF1 are no exceptions and highly specific inhibitors have recently been developed for both factors. Researchers had already designed potent HDAC inhibitors to manipulate histone acetylation in cancer patients but development of selective HAT inhibitors has been significantly more challenging (Wu et al., 2020). The MYST domain is extremely conserved between all family members, which has made it hard to design inhibitors specific for each HAT. Remarkably, two novel HAT inhibitors, WM-8014 and WM-1119, have been shown to be MOZ/MORF specific (Baell et al., 2018). Precise structural studies with MOZ acetyl-CoA binding pocket showed the inhibitors reversibly compete with acetyl-CoA for binding, thus preventing histone acetylation. *In vitro* inhibition studies revealed that both inhibitors were approximately 100 times more effective against MOZ and MORF when compared with other HATs. Moreover, they were the first HAT inhibitors showing nanomolar range of biochemical inhibition. *In vivo* studies showed that the inhibitors are able to prevent cancer growth in a zebrafish model of HCC as well as lymphoma in mice (Baell et al., 2018). Mechanistically, inhibition of MOZ activity leads to cellular senescence. These inhibitors have the potential to be powerful tools to study and treat MOZ-related cancers described in the previous section.

Epigenetic factors containing bromodomains are seen as potential drug targets because efficient and specific inhibition of bromodomain-containing BET proteins like Brd4 has been achieved and shown great anticancer effects in preclinical studies (Perez-Salvia and Esteller, 2017). BRPF1 is one of the three highly conserved BRPF family of proteins, harbouring a histone acetylation-binding bromodomain (Lloyd and Glass, 2018). Although the protein structures are similar, specific BRPF1 inhibitors have been developed. Benzoimidazolone based PFI-4, OF-1 and GSK6853 are BRPF1-specific inhibitors which prevent the protein to bind acetylated histones, affecting chromatin binding affinity (Bamborough et al., 2016; Meier et al., 2017). Studies with bone marrow cells and human primary monocytes showed that PFI-4 and OF-1 repress the transcription of osteoclastogenesis-related factors (Meier et al., 2017). The inhibitors do not affect normal cell growth or proliferation, which implies no high cytotoxic properties. Recent studies with GSK5959 (GSK6853 ortholog) have shown that BRPF1 inhibition can prevent HCC progression in cells and in mice, making these inhibitors possible therapeutic drugs for HCC treatment (Cheng et al., 2021). Another promising avenue is to develop synthetic lethality approaches targeting BRPF bromodomain in parallel to metabolism pathways producing acetyl-CoA, for example the GLUT1 glucose transporter as shown in breast cancer cells (Wu et al., 2019).

## Conclusion

A large amount of information has been gathered on the biochemical properties of the MOZ histone acetyltransferase complex. But precise *in vivo* molecular mechanisms are still largely not clearly understood in terms of recruitment and the function of the abundant H3K23ac mark deposited by the complex. It has become clear that functional studies need to consider the whole protein complex instead of just focusing on the HAT enzyme, as BRPF1 is essential for its activity on chromatin and contains several reader domains for epigenetic marks. The MOZ-BRPF1 complex plays crucial roles in cellular homeostasis, development and different human pathologies. The efficient design of specific drugs targeting MOZ and BRPF1 provides great promise to more precisely dissect their function *in vivo* but also develop powerful therapeutic avenues to treat patients.
